# Amplifying Photochromic Response in Tungsten Oxide Films with Titanium Oxide and Polyvinylpyrrolidone

**DOI:** 10.3390/nano14131121

**Published:** 2024-06-29

**Authors:** Min-Sung Kim, Jun-Ho Yoon, Hong-Mo Kim, Dong-Jun Lee, Tamaki Hirose, Yoshihiko Takeda, Jae-Pil Kim

**Affiliations:** 1 Lab of Organic Photo-Functional Materials, Department of Materials Science and Engineering, Seoul National University, 1 Gwanak-ro, Gwanak-gu, Seoul 08826, Republic of Korea; kms619@snu.ac.kr (M.-S.K.); junho0905@snu.ac.kr (J.-H.Y.); dongjunl@snu.ac.kr (D.-J.L.); 2 Semiconductor Analysis Team, Advanced Institute of Convergence Technology, 145 Gwanggyo-ro, Yeongtong-gu, Suwon-si 16229, Republic of Korea; hmkim0118@snu.ac.kr; 3 Hydrogen Related Materials Group, Research Center for Energy and Environmental Materials, National Institute for Materials Science (NIMS), Tsukuba 305-0003, Japan; hirose.tamaki.gp@alumni.tsukuba.ac.jp

**Keywords:** tungsten oxide nanoparticles, hybrid composite, dispersibility, photochromic property

## Abstract

Tungsten oxide (WO_3_) is known for its photochromic properties, making it useful for smart windows, displays, and sensors. However, its small bandgap leads to rapid recombination of electron–hole pairs, resulting in poor photochromic performance. This study aims to enhance the photochromic properties of WO_3_ by synthesizing hexagonal tungsten oxide via hydrothermal synthesis, which increases surface area and internal hydrates. Titanium oxide (TiO_2_) was adsorbed onto the tungsten oxide to inject additional charges and reduce electron–hole recombination. Additionally, polyvinylpyrrolidone (PVP) was used to improve dispersion in organic solvents, allowing for the fabrication of high-quality films using the doctor blade method. Characterization confirmed the enhanced surface area, crystal structure, and dispersion stability. Reflectance and transmittance measurements demonstrated significant improvements in photochromic properties due to the composite structure. These findings suggest that the introduction of TiO_2_ and PVP to tungsten oxide effectively enhances its photochromic performance, broadening its applicability in various advanced photochromic applications.

## 1. Introduction

Tungsten oxide (WO_3_) is an inorganic material with photochromic properties, characterized by high photostability and thermal stability, low toxicity, and ease of synthesis [1,2]. Due to these properties, it holds the potential for applications in various fields such as smart windows, displays, optical devices, and sensors [3,4]. Specifically, its photochromic properties, which cause a color change when exposed to light, offer advantages over thermochromic or electrochromic materials as it does not require additional energy sources or complex structures [4,5,6]. This makes it environmentally friendly and highly applicable, leading to extensive research and diverse exploration of its applications [2].

However, tungsten oxide has a smaller bandgap compared to other photochromic inorganic materials like ZnO, MoO_3_, and AgCl [7,8]. This small bandgap allows the easy recombination of electron–hole pairs generated by light, resulting in poor photochromic properties and limiting its applicability [2,4]. To overcome these drawbacks, research has focused on synthesizing tungsten oxide as quantum dots through self-assembly to increase the bandgap or modify the surface structure to enhance reactivity [5,7]. Additionally, methods such as doping with various metal particles to generate free electrons or combining with organic materials to enhance photochromic properties by introducing additional elements like electrons or protons have been studied [9,10,11].

The widely accepted photo-induced coloration mechanism of tungsten oxide can be explained as follows [12,13]:

(1) $${\mathrm{WO}}_{3}+h\nu \;\to \;{\mathrm{WO}}_{3}^{*}+e^{-}+h^{+}$$

(2) $$2h^{+}+H_{2}O\;\to \;2H^{+}+O$$

(3) $${\mathrm{WO}}_{3}+{\mathrm{xH}}^{+}+xe^{-}\;\to \;H_{x}{W^{6+}}_{1-x}{W^{5+}}_{x}O_{3}$$

When UV light irradiates WO_3_, the electrons generated by the light move to the conduction band, while holes are created in the valence band (reaction (1)). The holes in the valence band react with the -OH groups of water molecules to produce protons and oxygen radicals (reaction (2)). Finally, the electrons that moved to the conduction band combine with W^6+^ to reduce it to W^5+^, and these electrons, along with the previously generated protons, form hydrogen tungsten bronze (H_x_W^6+^_1−x_W^5+^_x_O_3_) which has a blue color (reaction (3)). This mechanism demonstrates that the presence of e–h pairs and protons can significantly enhance the photochromic properties [14,15].

Enhancing photochromic properties within the photochromic mechanism can be achieved by generating more electron–hole pairs induced by light or producing more protons. In this study, we synthesized tungsten oxide with a hexagonal structure containing hydrate through hydrothermal synthesis. This hexagonal structure, with both hexagonal and trigonal channels, has a larger surface area compared to the monoclinic structure of tungsten oxide, which only has trigonal channels [16,17,18]. This increased surface area enhances reactivity, resulting in improved photochromic properties [18,19,20]. Additionally, the internal structure containing hydrate can supply more protons, further enhancing photochromic properties due to the combined effect of increased surface area and proton availability.

We then introduced a structure where titanium oxide is adsorbed onto the synthesized tungsten oxide to inject more charges. Titanium oxide is highly reactive to light, stable, and possesses high optical transparency, making it useful in batteries, solar cells, and photocatalysts [21,22]. When titanium oxide is introduced to tungsten oxide, it allows the absorption of a wider range of light wavelengths. The large bandgap of titanium oxide (approximately 3.2 eV for the anatase phase) plays a crucial role in reducing the recombination of electron–hole pairs when incorporated with tungsten oxide. When TiO_2_ is exposed to light, it generates electron–hole pairs. The photo-induced electrons can transfer from TiO_2_ to WO_3_, reducing the recombination rate by spatially separating the charge carriers. This separation enhances the photochromic properties of the composite material [23,24]. This process ensures that more photogenerated electrons participate in the photochromic reaction, ultimately enhancing the overall photochromic properties. By incorporating TiO_2_ with WO_3_, the composite material exhibits an improved photochromic response, demonstrating the effectiveness of this approach.

We inject electrons into tungsten oxide, which is a critical element of this photochromic mechanism, and choose the doctor blade method (solution-based process) to produce films with excellent photochromic properties without the need for vacuum equipment, unlike conventional methods such as CVD, ALD, and sputtering. To achieve this, tungsten oxide particles need to be well dispersed in the solvent. Typically, metal oxide particles do not disperse well in organic solvents, leading to particle aggregation or precipitation, which degrades the quality of solution-based films. To overcome this issue, organic ligands that act as dispersants can be adsorbed onto the particle surface to form an ion layer that increases repulsion between particles, or hydrophilic and lipophilic ligands can be used to improve mixing with the solvent [11,16,20].

Finally, to overcome the aforementioned drawbacks of tungsten oxide, titanium oxide was introduced to inject more electrons into tungsten oxide and enhance its photochromic properties. Additionally, to improve dispersion in the solvent for solution-based film fabrication, polyvinylpyrrolidone (PVP) was introduced into the particles. This structure allows more electrons generated by light to be injected into tungsten oxide through the junction with titanium oxide and, the introduction of PVP increases repulsion between particles, minimizing aggregation [25].

## 2. Materials and Methods

### 2.1. Materials

Ammonium tungstate pentahydrate (ATP, 5(NH_4_)_2_O∙12WO_3_∙5H_2_O), polyvinylpyrrolidone (PVP, (C_6_H_9_NO)_n_), and anhydrous oxalic acid (98%) were sourced from Alfa Aesar. PGME (extra pure, 1-Methoxy-2-propanol, 98.5%) and TiO_2_ (P25) were obtained from SAMCHUN Pure Chemical Co., Ltd. (Daegu, Republic of Korea). All chemicals were utilized directly without any additional purification.

### 2.2. Preparation of WO_3_ Nanoparticles

WO_3_ nanoparticles were synthesized through a hydrothermal process. The steps were as follows: In a 150 mL reactor, 15.6 g of ATP was dissolved in 70 mL of distilled water in a beaker. To this, 20 mL of oxalic acid solution (prepared by dissolving 10 g of oxalic acid in 100 mL of deionized water) was added while stirring, and the pH was adjusted to 1 using 2 M HCl. The mixture was stirred for 4 h before being transferred to a reactor and heated at 120 °C for 12 h. Post-reaction, the resulting tungsten oxide was separated via centrifugation and the precipitate was washed twice with ethanol.

### 2.3. Synthesis of the Composite

Following the synthesis of the nanoparticle composite, it underwent two ethanol and deionized water washes each. TiO_2_ was then added in molar ratios of 3%, 5%, and 10%, respectively, and stirred for 3 h. PVP was incorporated into the tungsten oxide–ethanol mixture at 50 wt%, and this mixture was stirred for 1 h, then ultrasonicated for 30 min. The process concluded with a 12 h stirring period. The final product was centrifuged and washed with PGME, then mixed with 20 wt% PGME solvent to achieve a dispersed solution.

### 2.4. Fabrication of Photochromic Film

A glass slide was cleaned using nitrogen gas. A solution containing 30 wt% tungsten oxide in 1-methoxy-2-propanol (PGME) was combined with 20 wt% acrylate binder (3:2 ratio) and stirred for 24 h at room temperature. This solution was then drop-casted onto the glass slide and coated using a doctor blade technique. The sample was heated at 100 °C for 2 min to remove any residual solvent.

### 2.5. Characterization and Measurement

UV-Vis reflectance spectra were recorded with a JASCO V-670 and V-770 spectrophotometer (Tokyo, Japan). Fourier-transform infrared (FT-IR) spectroscopy was performed using a Bruker TENSOR27 spectrometer (Billerica, MA, USA). UV-Vis transmittance spectra were obtained with a Perkin Elmer Lambda 1050 spectrophotometer (Waltham, MA, USA). Field emission scanning electron microscopy (FE-SEM) images were taken with a JEOL JSM-7800F Prime instrument (Tokyo, Japan), with Pt coating using a Zeiss MERLIN Compact (Oberkochen, Germany). High-resolution X-ray diffraction (HRXRD) of the coated film was examined using a SmartLab instrument (Tokyo, Japan) with Cu–Kα X-rays (λ = 0.154 nm). Field emission transmission electron microscopy (FE-TEM) images were captured using a JEOL JEM-F200 instrument (Tokyo, Japan). Dynamic light scattering (DLS) measurements were conducted with a DLS-8000HAL, and zeta potential analysis with an ELSZ-1000, both from Photal Otsuka Electronics (Osaka, Japan), with 1 min measurements and auto-fitting of the correlation function using Anton Paar’s Kalliope software (304813). Thermogravimetric analysis (TGA) was performed at a heating rate of 10 °C/min using a TA Instruments SDT Q600 (New Castle, DE, USA). X-ray photoelectron spectroscopy (XPS) was conducted with a Thermo Fisher Scientific Sigma Probe for electron spectroscopy chemical analysis (Waltham, MA, USA).

## 3. Results and Discussion

### 3.1. Characterization of the Synthesized Tungsten Oxide

**Figure 1:**
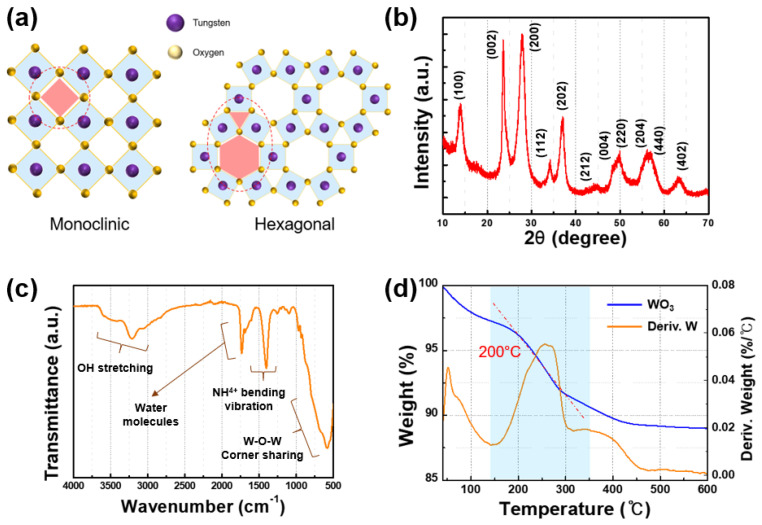
(**a**) Monoclinic and hexagonal structure of tungsten oxide, (**b**) X-ray diffraction of synthesized tungsten oxide, (**c**) FT-IR analysis, and (**d**) TGA analysis.

Following the outlined synthesis procedure utilized in our previous study, we successfully prepared hexagonal WO_3_ particles exhibiting photochromic properties through a hydrothermal method [25]. The hexagonal crystalline structure of tungsten oxide, as illustrated in Figure 1a, is integral to its photochromic behavior. Unlike the commercially available monoclinic form of tungsten oxide, which contains only trigonal channels, the hexagonal variant features both trigonal and hexagonal channels. This dual-channel structure results in an increased surface area, enhancing reactivity and facilitating electron mobility, thus significantly improving the photochromic properties.

To examine the crystal structure of the synthesized tungsten oxide, we performed XRD measurements. As depicted in Figure 1b, the principal peak corresponds to the hexagonal structure (JCPDS card no. 33-1387) [14,26,27,28]. Hexagonal tungsten oxide crystals feature a three-dimensional framework of corner-sharing octahedral interconnected by oxygen atoms. The bonding characteristics of the synthesized tungsten oxide were validated using Fourier-transform infrared (FT-IR) spectroscopy. In Figure 1c, a vibration peak observed around 700 cm⁻¹ is indicative of the bridging vibration of corner-sharing octahedra (W–O_inter_–W) in the WO_3_ structure. This mode demonstrates the oxygen atoms being shared between neighboring tungsten atoms. Additionally, peaks detected around 1600 cm^−1^ suggest the presence of intercalated water molecules, which are involved in W–O∙∙∙H_2_O interactions. Peaks near 3450 cm^−1^ are associated with the in-plane bending vibrations of W–OH, implying that oxygen vacancies or single bonds on the particle surface are bonded as hydroxyl groups (OH). The sharp peak at approximately 1400 cm^−1^ can be attributed to the absorption of NH^4+^ ions from the precursor material [28]. These findings indicate that the synthesized tungsten oxide predominantly consists of W–O bonds, which indirectly confirm the octahedral structure facilitated by oxygen sharing. Moreover, the presence of OH groups as single bonds or oxygen vacancies on the surface is clearly identified.

Figure 1d illustrates the TGA analysis of the synthesized tungsten oxide, revealing its hydration level. Organic materials adsorbed on the surface begin to decompose at temperatures up to approximately 100 °C, and a 7.5% hydrate decomposition occurs between approximately 150 °C and 250 °C. These findings confirm the octahedral structure of the synthesized tungsten oxide and indicate the presence of water molecules within the framework.

**Figure 2:**
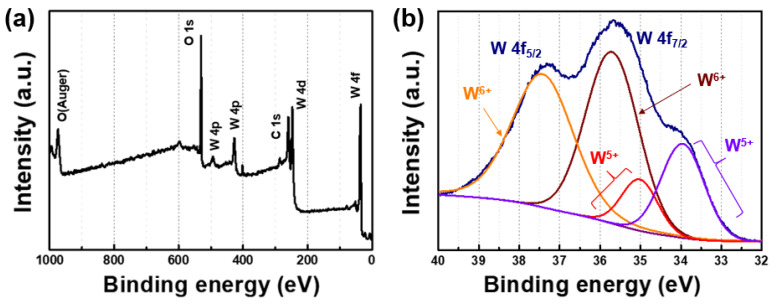
(**a**) Survey XPS spectra of synthesized tungsten oxide and a (**b**) high-resolution W 4f spectrum of the same sample.

Furthermore, by examining the XPS in Figure 2, it can be observed that the tungsten oxide synthesized via hydrothermal synthesis primarily forms a 6+ oxidation state and exhibits a structure with oxygen vacancies. The main peaks in the XPS spectrum correspond to W 4f at 35.7 eV, W 4d at 280.5 eV, C 1s at 285.2 eV, and O 1s at 532.2 eV. The remaining residuals from the synthesis of carbon and nitrogen can be identified, and slight peaks are still present even after rinsing. High-resolution XPS peaks reveal the existence of 4f_5/2_ at 38.5 eV and 4f_7/2_ at 35.5 eV. Upon examining the split peaks, W^6+^ 4f_7/2_ at 35.3 eV and W^6+^ 4f_5/2_ at 37.5 eV can be identified, while weak binding energy peaks correspond to W^5+^ at 34.5 eV and W^5+^ at 36.5 eV.

**Figure 3:**
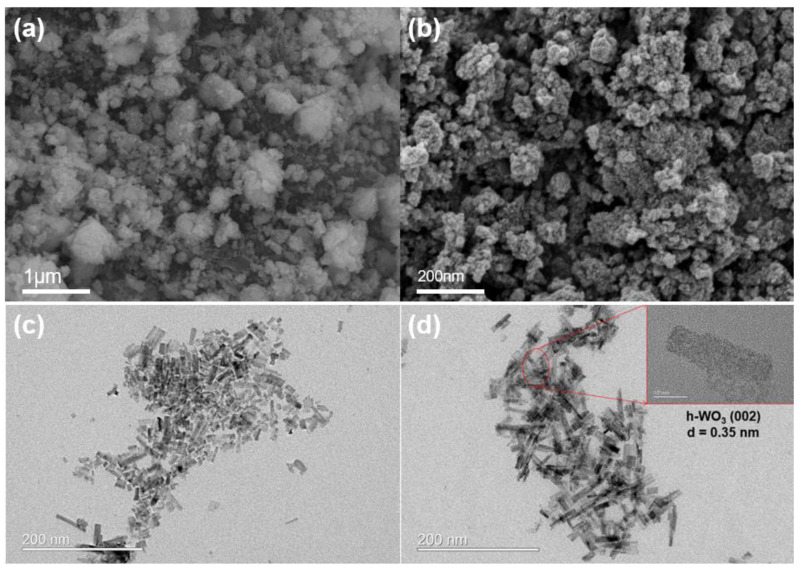
(**a**,**b**) SEM image of synthesized WO_3_ and (**c**,**d**) HRTEM image. The inset of (**d**) shows lattice parameters of 0.35 nm (002) and plate-like WO_3_ morphology.

To examine the morphology and microstructure of the synthesized tungsten oxide particles, SEM and TEM analyses were conducted. In Figure 3a,b, SEM images reveal that the synthesized tungsten oxide particles exhibit agglomeration ranging in size from approximately 100 nm to several micrometers. TEM images in Figure 3c,d show plate-like shapes with dimensions of approximately 40 nm. High-resolution TEM images indicate a lattice parameter of 0.35 nm for the (002) lattice and 0.67 nm for the (100) lattice [29,30].

### 3.2. Characterization of Tungsten Oxide with Titanium Oxide and Polyvinylpyrrolidone

The synthesized tungsten oxide possesses a hexagonal structure, which provides a wide surface area and contains internal hydrates that enhance its photochromic properties. However, compared to other photochromic inorganic materials, tungsten oxide faces the challenge of rapid recombination of photo-induced electron–hole pairs, which leads to lower photochromic performance. To address these drawbacks, we adsorbed titanium oxide onto the tungsten oxide. This approach injects additional charges, thereby reducing electron–hole recombination and enhancing the overall photochromic properties of the material.

**Figure 4:**
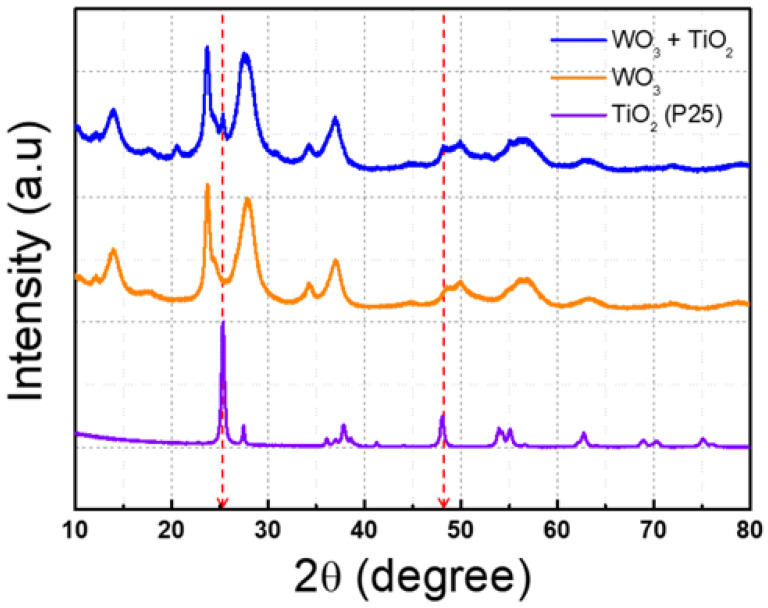
X-ray diffraction of synthesized tungsten oxide and titanium oxide. The orange line is tungsten oxide, the violet line is titanium oxide, and the blue line is tungsten oxide with titanium oxide. (The red dotted line indicates the TiO_2_ confirmation line).

To confirm the adsorption of titanium oxide onto tungsten oxide, XRD and TEM analyses were conducted. In Figure 4, the 2θ values of 25.31 for the anatase (101) peak and 27.4 for the rutile (110) peak of P25 titanium oxide can be observed, indicating the presence of adsorbed material of tungsten oxide and titanium oxide in the XRD peak of the blue line [31,32].

**Figure 5:**
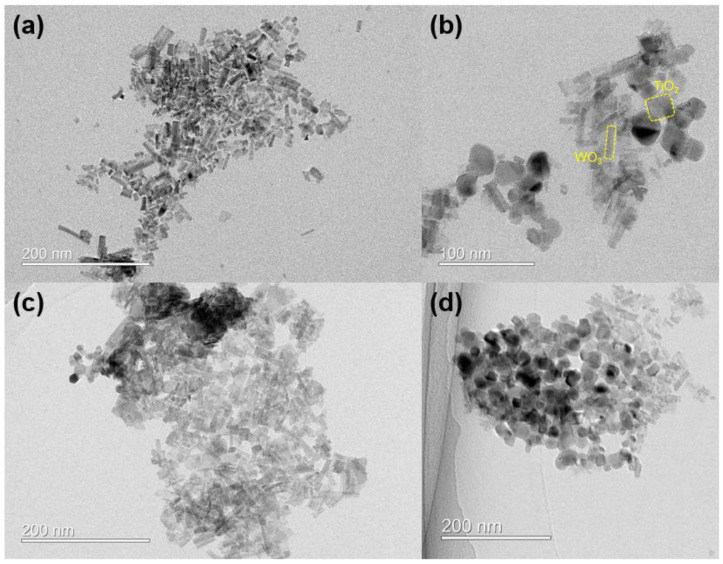
HRTEM image of tungsten oxide with titanium oxide. (**a**) Only WO_3_, (**b**) addition of 3% TiO_2_, (**c**) 5% TiO_2_, and (**d**) 10% TiO_2_ mole fraction. For TiO_2_, it has a spherical square shape with dimensions of approximately 20 nm. WO_3_ has a plate-like shape with dimensions of approximately 10 nm in width and 30 nm in length.

TEM analysis was conducted to investigate the surface structure of tungsten oxide with adsorbed titanium oxide particles. Typically, the conventional method for creating a junction between tungsten oxide and titanium oxide involves co-synthesizing them as precursors. However, it was observed that co-synthesis led to the formation of many residual materials and aggregated particles after synthesis, resulting in the generation of numerous particles on the film surface when films were produced (Figure S1 of Supplementary Materials). Therefore, we synthesized tungsten oxide first and then adsorbed TiO_2_ particles (P25) onto it. Figure 5 shows the structure according to the mole fraction of titanium oxide introduced into tungsten oxide. First, in Figure 5a, it can be observed that the hexagonal structure of tungsten oxide synthesized through hydrothermal synthesis has a plate-like structure with dimensions of approximately 10 nm in width and 30 nm in length. Figure 5b–d demonstrate the adsorption patterns according to the mole fraction of titanium oxide introduced. Particles with a mole fraction of 3% titanium oxide exhibit an appropriate adsorption ratio between tungsten oxide and titanium oxide. However, particles with 5% and 10% mole fractions show excessive coverage of tungsten oxide by titanium oxide particles. Therefore, exceeding the optimal input amount leads to a decrease in the amount of light reaching tungsten oxide, resulting in a reduction in photochromic properties and aggregation of particles on the film surface, ultimately degrading the properties of the photochromic film (Figure S3 of Supplementary Material) [32,33,34]. These findings are consistent with subsequent verification of reflectance analysis.

**Figure 6:**
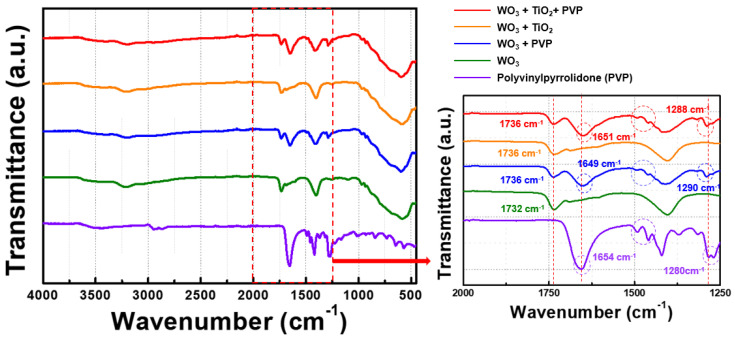
FT-IR analysis of tungsten oxide composites. The left image is the overall range of each material and the right image is an enlarged comparison of the range from 1250 to 2000 cm^−1^.

To produce films using the doctor blade method with tungsten oxide particles adsorbed with titanium oxide, it is essential to enhance the dispersibility in the solvent. Typically, metal oxide particles poorly disperse and tend to aggregate or precipitate in organic solvents, resulting in large particles on the film surface during film production [11,35,36]. To address this issue, polyvinylpyrrolidone was introduced as a dispersant. FT-IR analysis was conducted to confirm this combination. In Figure 6, when polyvinylpyrrolidone is adsorbed onto synthesized tungsten oxide, shifted peaks are observed. Specifically, when polyvinylpyrrolidone is bound to tungsten oxide or tungsten oxide/titanium oxide particles, the main peak of polyvinylpyrrolidone, the C=O stretching vibration, shifts to 1649 cm^−1^ and 1651 cm^−1^, and the C–N vibrations peak shifts to 1290 cm^−1^ and 1288 cm^−1^. These shifts suggest that polyvinylpyrrolidone is adsorbed onto and interacts with the surface of tungsten oxide, possibly onto hydrogen or oxygen vacancies, rather than forming a new bonding structure chemically [37,38].

### 3.3. Dispersibility Analysis in Organic Solvents for Solution-Based Film Fabrication

To assess the dispersion of the synthesized tungsten oxide composite, zeta potential analysis was conducted. Zeta potential analysis is a method to evaluate the dispersion state and stability of particles by measuring the potential of the charged layers on the particle surface. Typically, metal oxide particles are considered to be stably dispersed in a solvent when the zeta potential is approximately 30 mV [39,40]. Values below 30 mV indicate insufficient repulsion among particles, leading to aggregation and unstable dispersion, while values above 30 mV indicate sufficient repulsion and stable particle distribution.

**Figure 7:**
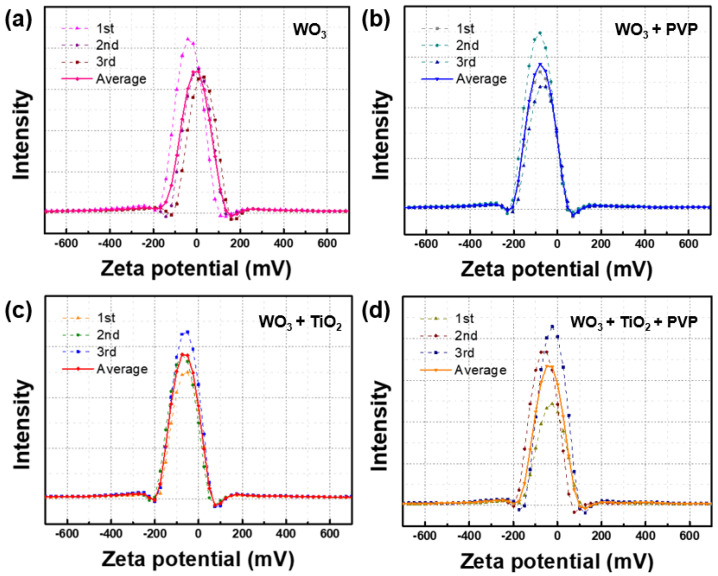
Zeta potential analysis of WO_3_ and composites. (**a**) Only WO_3_, (**b**) WO_3_ with polyvinylpyrrolidone, (**c**) WO_3_ with TiO_2_, and (**d**) final composite (WO_3_ with TiO_2_ capsulated by polyvinylpyrrolidone).

Samples were dispersed in solvent through sonication for approximately 10 min before measurement. Upon examination of Figure 7 and Table 1, it was observed that tungsten oxide particles without the dispersing agent, PVP, and tungsten oxide/titanium oxide composite particles exhibited zeta potentials of 28.8 mV and 28.4 mV, respectively, indicating relatively unstable states [41,42]. However, with the introduction of PVP, the zeta potentials increased to 70.2 mV and 68.3 mV, respectively, demonstrating stable dispersion.

**Figure 8:**
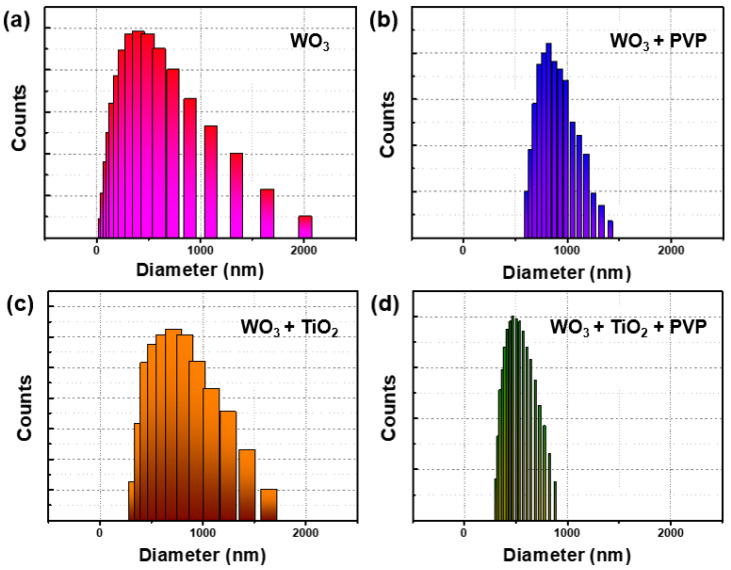
DLS analysis of WO_3_ and composites. (**a**) Only WO_3_, (**b**) WO_3_ with PVP, (**c**) WO_3_ with TiO_2_, and (**d**) final composite.

Additionally, dynamic light scattering (DLS) analysis was conducted using the same dispersion solution to further evaluate dispersibility [43,44]. As depicted in Figure 8 and Table 2, without PVP, the size distribution showed significant heterogeneity, with particle sizes ranging from 2800 nm to 1284 nm. Conversely, with the addition of PVP, the particle sizes narrowed to 800 nm and 900 nm, indicating improved dispersion. The distribution histogram confirmed that both tungsten oxide and titanium oxide composite particles exhibited narrower dispersion widths when PVP was introduced (Figure S4 of Supplementary Materials).

These results suggest that PVP adsorbs onto the surface of tungsten oxide particles, imparting a negative charge, increasing interparticle repulsion, preventing aggregation, and enhancing dispersion in the solvent [45,46,47].

### 3.4. Reflectance for Confirming Enhanced Photochromic Properties

**Figure 9:**
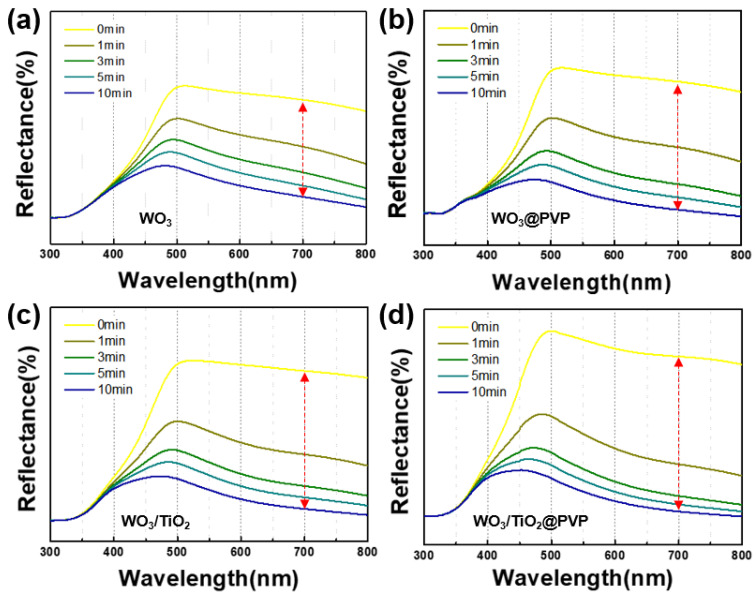
Reflectance of tungsten oxide composites (The red line indicates the total change observed over 10 min). (**a**) Only WO_3_, (**b**) WO_3_@PVP, (**c**) WO_3_/TiO_2_, and (**d**) final composite (WO_3_/TiO_2_@PVP). (The y-axis is the reflectance value of 0 to 90%.)

To quantitatively assess the alterations in photochromic properties of the synthesized tungsten oxide composite, reflectance spectroscopy was conducted on the powdered form. Using UV radiation (VL-6.LC, 365 nm tube, 50/60 Hz), each material was subjected to irradiation for 1, 3, 5, and 10 min durations while monitoring the reflectance changes. The resultant data, as illustrated in Figure 9 and summarized in Table 3, delineate the spectral changes observed at 700 nm following 1 min UV irradiation.

Monoclinic tungsten oxide exhibited a reflectance change of 20.1%, whereas the tungsten oxide/PVP composite demonstrated 27.9% variation. Conversely, tungsten oxide adorned with titanium oxide showcased a 30.8% shift, while the final tungsten oxide/titanium oxide/PVP composite displayed a substantial change of 42.2%.

The alterations reveal a significant enhancement in the photochromic properties due to the combined effects of titanium oxide (TiO_2_) and polyvinylpyrrolidone (PVP) on tungsten oxide (WO_3_). The incorporation of TiO_2_ introduces additional electron pairs into WO_3_, thereby augmenting its photochromic attributes. This enhancement is particularly notable due to the narrow bandgap of WO_3_, which typically facilitates electron–hole pair recombination, thus reducing its photochromic efficiency. By increasing the electron population, which is crucial for the photochromic mechanism, the overall photochromic properties are markedly improved.

Moreover, the high electron mobility inherent in TiO_2_ facilitates the migration of electron–hole pairs to the WO_3_ matrix. When TiO_2_ adsorbs onto the WO_3_ surface, it results in an expansion of the surface area, providing a greater number of reactive sites for photon-induced reactions. This process not only catalyzes the photochromic mechanism within WO_3_ but also causes substantial chromatic changes due to enhanced hydrogen interactions.

Furthermore, the addition of PVP plays a dual role by enhancing the dispersibility of WO_3_ particles in the solvent and inducing the ligand-to-metal charge transfer (LMCT) effect, which amplifies the photochromic properties. (Figure S2 of Supplementary Materials) [48]. The LMCT effect involves the transfer of charge from the ligand species adsorbed on the particle surface to the metal oxide. For PVP, this charge transfer occurs as the non-bonding electron pairs from nitrogen atoms transition to WO_3_, strengthening the photochromic mechanism.

Additionally, the presence of hydrogen in PVP facilitates the generation of protons, thereby improving the overall electron–proton injection balance required for robust photochromic mechanisms. This improvement is evident from reflectance measurements, which show significant alterations following the introduction of TiO_2_ and PVP. These combined effects lead to a substantial enhancement in the photochromic properties of WO_3_ particles, thus significantly improving photochromic efficiency.

### 3.5. Photochromic Properties in Film

**Figure 10:**
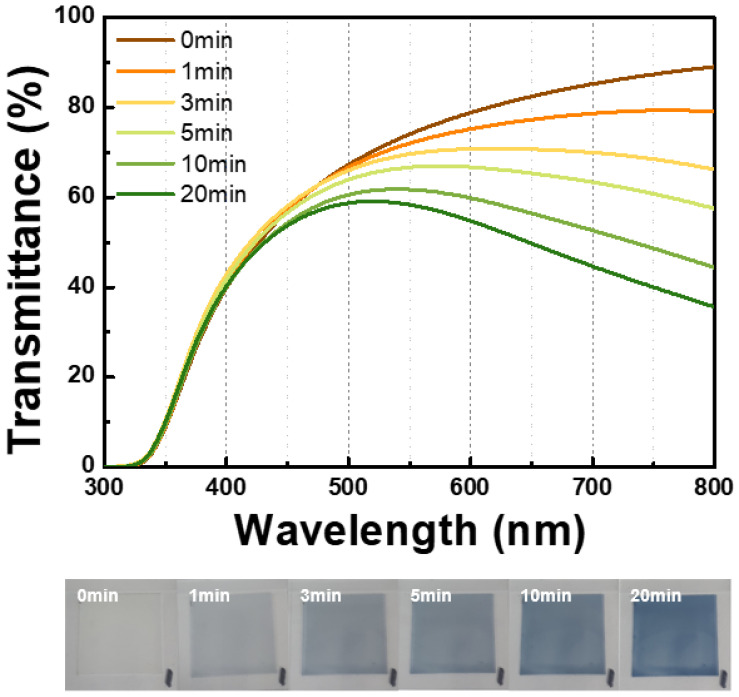
Transmittance of the photochromic film over time. Maximum coloration time is 20 min.

Finally, the synthesized tungsten oxide composite was dispersed in PGME solvent at 20 wt%, mixed with an acrylate binder in a ratio of 3:2 (wt%), and stirred for approximately 3 h to ensure thorough blending. Subsequently, the film was fabricated onto a glass substrate using the blade coating method. The coated film was baked at approximately 100 °C for 2 min. The film thickness, 4 to 5 µm, was confirmed through cross-sectional SEM measurements (Figure S5 of Supplementary Materials). Utilizing the same UV lamp specifications employed in the reflectance analysis, measurements were conducted at various time intervals, up to a maximum duration of 20 min (Figure 10). When compared at a wavelength of 700 nm, the film exhibited an initial transmittance of 85.2%, which decreased to 44.7% at the maximum value of 20 min, indicating a variation of approximately 40.5% (Table 4). Compared to the WO_3_@PVP composite film without TiO_2_, we confirmed that the introduction of TiO_2_ resulted in more than double the photochromic performance (Figure S6 of Supplementary Materials). This enhanced photochromic property aligns with the improved reflectance results observed earlier.

In summary, we synthesized hexagonal structured tungsten oxide via hydrothermal synthesis, confirming its potential for enhanced photochromic properties owing to its larger surface area compared to conventional monoclinic structures and the presence of hydrates. Furthermore, by introducing titanium oxide to alleviate the drawback of low photochromic properties attributed to the recombination of electron–hole pairs due to a small bandgap, and incorporating PVP to serve both as a dispersant and as a means to inject electrons and protons into the tungsten oxide, we successfully synthesized a tungsten composite film with excellent photochromic properties. Ultimately, the synergistic effects of titanium oxide and PVP resulted in the fabrication of an outstanding photochromic film.

## 4. Conclusions

In this study, we synthesized a tungsten oxide composite with exceptional photochromic properties and dispersibility by introducing titanium oxide and polyvinylpyrrolidone (PVP). Notably, the tungsten oxide material obtained through hydrothermal synthesis exhibited a hexagonal structure and included hydrate, further enhancing its photochromic properties. Utilizing a solution-based doctor blade method, we successfully fabricated superior photochromic films without the need for complex processing steps. Through dynamic light scattering (DLS) and zeta potential analysis, we ensured excellent dispersion of tungsten oxide/titanium oxide particles in organic solvents. Additionally, reflectance measurements of the powder state confirmed enhanced photochromic properties through electron injection and proton donation. When fabricating the films, the introduction of titanium oxide for electron injection and PVP for ligand-to-metal charge transfer (LMCT) effects and proton donation resulted in more than a twofold enhancement in photochromic properties compared to before the introduction of these materials. These findings emphasize the crucial role of PVP ligand interactions in enhancing the photochromic properties of tungsten oxide composites. In conclusion, the synthesized tungsten oxide hybrid composite, featuring a hexagonal structure and hydrate inclusion, represents a significant advancement in photochromic material research. These results offer promising prospects for the environmentally friendly and practical application of photochromic films, marking a departure from complex processing steps and high-cost film fabrication methods.

## Figures and Tables

**Table 1:** Zeta potential analysis of the tungsten oxide composite. Each material was measured in five repeat measurements, with three times readings per measurement, to calculate the average value.

Material	WO_3_	WO_3_@PVP	WO_3_/TiO_2_	WO_3_/TiO_2_@PVP
Average zeta potential (mV)	28.8	70.2	28.4	68.3

**Table 2:** DLS data were collected through 10 measurements. Average particle size decreased when PVP was introduced and the dispersion showed a narrower distribution.

Material	WO_3_	WO_3_/PVP	WO_3_/TiO_2_	WO_3_/TiO_2_/PVP
Distribution range (nm)	48.8~2849	614.7~1417	361.3~1645.3	81.9~982.1
Size variation (nm)	2800.2	802.3	1284	900.2

**Table 3:** Reflectance was measured in powder form by introducing titanium oxide and polyvinylpyrrolidone to WO_3_. The reflectance variation is at a maximum value and 700 nm by UV radiation for 1 min.

Sample	Hexagonal WO_3_		h-WO_3_/PVP		h-WO_3_/TiO_2_		h-WO_3_/TiO_2_/PVP	
Reflectance	%R (max)	%R (700 nm)	%R (max)	%R (700 nm)	%R (max)	%R (700 nm)	%R (max)	%R (700 nm)
Initial state	68.0	62.0	75.6	69.7	76.2	70.7	84.1	79.2
UV 1 min	53.9	41.9	53.6	41.8	50.7	39.9	53.0	37.0
ΔR (%)	14.1	20.1	22.0	27.9	25.5	30.8	31.1	42.2

**Table 4:** Transmittance of the photochromic film over time (at 700 nm wavelength).

Time	0 min	1 min	3 min	5 min	10 min	20 min
Transmittance (%) at 700 nm	85.2	78.7	70.0	63.4	52.6	44.7
ΔT (%)	85.2	ΔT = 40.5				44.7

## Data Availability

Data are contained within the article and Supplementary Materials.

## Supplementary Materials

The following supporting information can be downloaded at https://www.mdpi.com/article/10.3390/nano14131121/s1: Scheme S1: The band gap potential diagram of the WO_3_/TiO_2_ composite; Figure S1: SEM images of the film surfaces by different methods for tungsten oxide and titanium oxide; Figure S2: The scheme of ligand to metal charge transfer (LMCT) effect; Figure S3: Color change depends on the amount of titanium oxide; Figure S4: HRTEM of tungsten oxide hybrid composite; Figure S5: The cross-sectional SEM image of the photochromic film; Figure S6: Transmittance of photochomic films. Reference [49] is cited in the Supplementary Materials.
