# Supplementary material for: Amplifying Photochromic Response in Tungsten Oxide Films with Titanium Oxide and Polyvinylpyrrolidone
Source: Nanomaterials (Basel). 2024 Jun 29;14(13):1121. doi: 10.3390/nano14131121 (PMC11243557; doi:10.3390/nano14131121)
Supplement: Supplementary file 1 [file nanomaterials-14-01121-s001.zip › nanomaterials-3055298-supplementary.pdf]

Supplementary Materials

Amplifying photochromic response in tungsten oxide films with titanium oxide and polyvinylpyrrolidone

Min-Sung Kim [1,†], Jun-Ho Yoon [1,†], Hong-Mo Kim [2], Dong-Jun Lee [1], Tamaki Hirose [3], Yoshihiko Takeda [3,*] and Jae-Pil Kim [1,*]

[1] Lab of Organic Photo-functional Materials, Department of Materials Science and Engineering, Seoul National University, 1 Gwanak-ro, Gwanak-gu, Seoul 08826, Republic of Korea; kms619@snu.ac.kr (M.S.K.); junho0905@snu.ac.kr (J.H.Y.); dongjunl@snu.ac.kr (D.J.L.)
[2] Semiconductor Analysis Team, Advanced Institute of Convergence Technology, 145 Gwanggyo-ro Yeongtong-gu, Suwon-si 16229, Republic of Korea; hmkim0118@snu.ac.kr (H.M.K.)
[3] Hydrogen Related Materials Group, Research Center for Energy and Environmental Materials, National Institute for Materials Science (NIMS), Tsukuba 305-0003, Japan; hirose.tamaki.gp@alumni.tsukuba.ac.jp (T.H.)
* Correspondence: takeda.yoshihiko@nims.go.jp (Y.T.); jaepil@snu.ac.kr (J.P.K.)
† These authors contributed equally to this work.

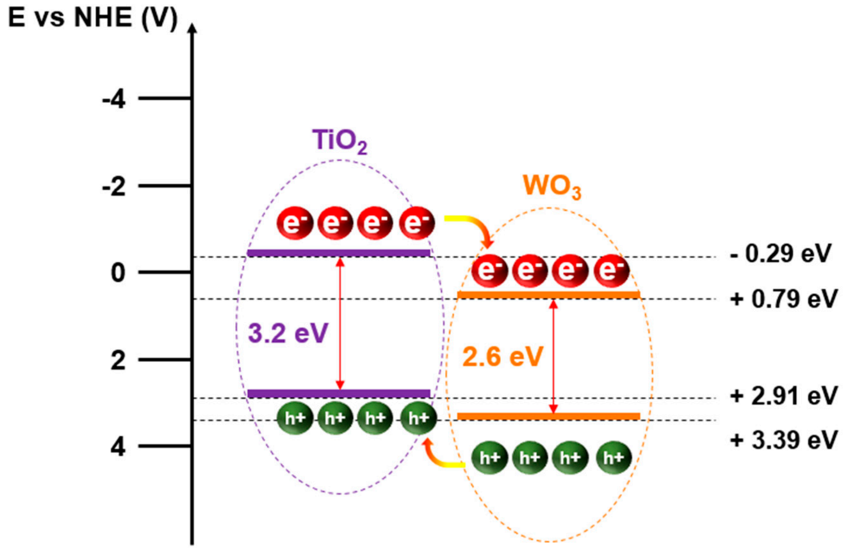

Scheme 1. The band gap potential diagram of the WO_3/TiO_2 composite.

To calculate the band edge positions of the heterostructure nanocomposite, the following equations were utilized:

$$E_{VB} = X - E_e + 0.5E_g \quad (1)$$

$$E_{CB} = E_{VB} - E_g \quad (2)$$

where X is the absolute electronegativity of the semiconductor (X for TiO_2 is 5.81 eV and X for WO_3 is 6.59 eV), E_e is the energy of free electrons on the hydrogen scale (approximately 4.5 eV), and E_g is the band gap energy of the semiconductor (E_g for TiO_2 is 3.2 eV and E_g for WO_3 is 2.6 eV). Based on these expressions, the calculated valence band (VB) and conduction band (CB) potentials of TiO_2 are 2.91 V and -0.29 V (versus NHE), respectively, while those of WO_3 are 3.39 V and 0.79 V (versus NHE).

The conduction band potential of WO_3 is lower than that of TiO_2, and the valence band edge of TiO_2 is higher than that of WO_3. Under UV light irradiation, electrons migrate from the CB of TiO_2 to the CB of WO_3, and holes transfer from the VB of WO_3 to the VB of TiO_2 [1].

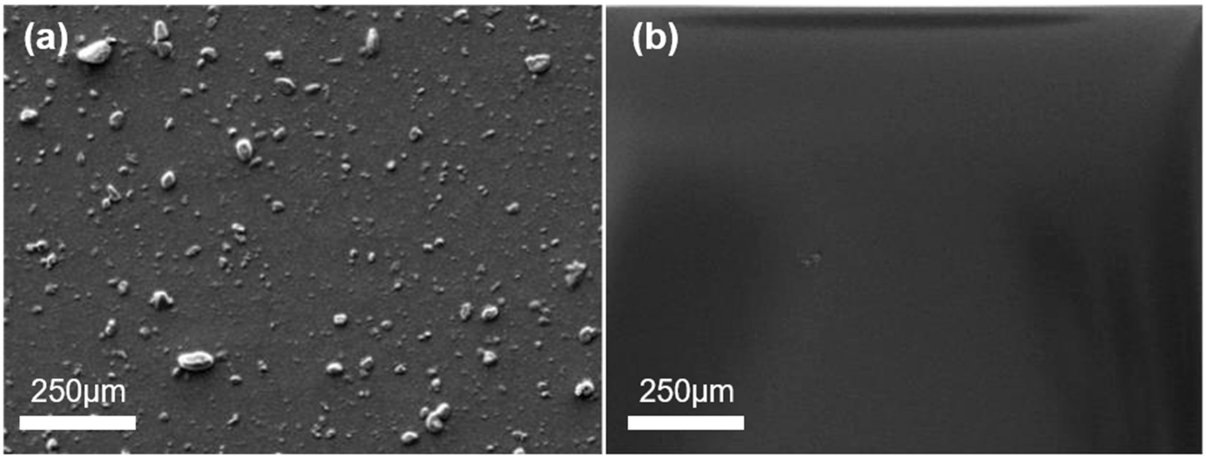

Figure S1. SEM images of the film surfaces by different methods for tungsten oxide and titanium oxide. (a) Film fabricated with particles synthesized by mixing tungsten oxide and titanium oxide precursors, and (b) film fabricated by adsorbing titanium oxide onto hydrothermally synthesized tungsten oxide. Particles synthesized from the precursor state are difficult to control in size, which leads to easy aggregation and results in large aggregated particles on the surface when fabricating films.

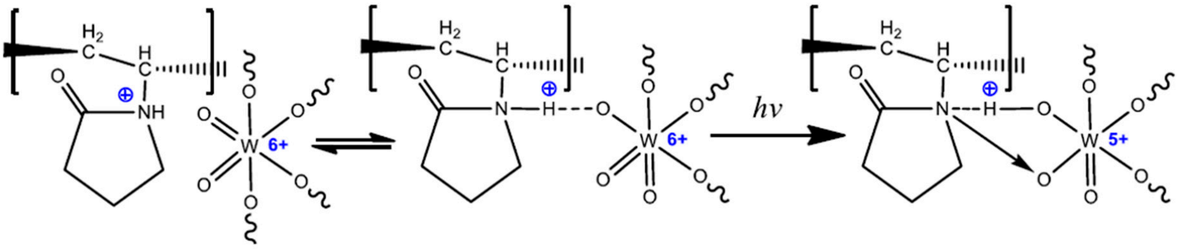

Figure S2. The scheme of ligand to metal charge transfer (LMCT) effect. The unshared electrons in the nitrogen atoms of polyvinylpyrrolidone (PVP) facilitate charge transfer to the tungsten oxide, thereby further enhancing the photochromic properties [2]. This effect not only improves the dispersion of the tungsten oxide composite due to PVP acting as a dispersant but also enhances the photochromic properties through charge transfer, resulting in a synergistic effect.

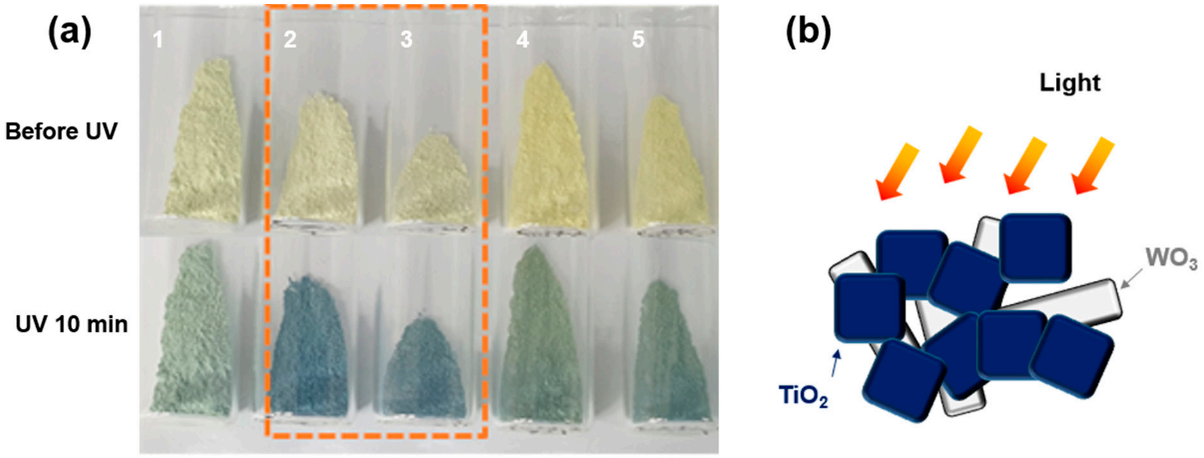

Sample #	1	2	3	4
Addition ratio of TiO_2 (P25)	0	3%	5%	10%

Figure S3. (a) color change depends on the amount of titanium oxide added, (b) the phenomenon where a large amount of titanium blocks the light reaching the tungsten oxide. If the amount of titanium oxide exceeds an optimal level, it screens the tungsten oxide from the light, resulting in reduced photochromic properties [3-6]. Consequently, in this study, we fixed the optimal addition

ratio at 3%. This was confirmed by TEM images, which show how much the titanium oxide particles cover the tungsten oxide particles.

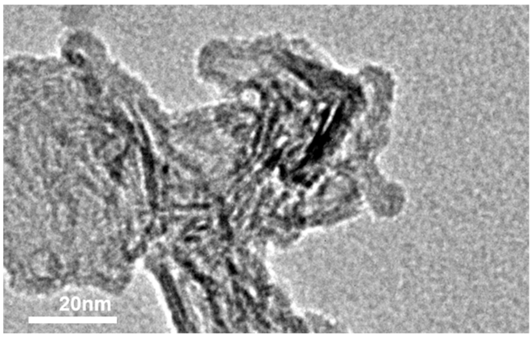

Figure S4. HRTEM of tungsten oxide hybrid composite. Tungsten oxide and titanium oxide composite were capsulated by PVP. By this structure, the composite particles are stable in the solution state.

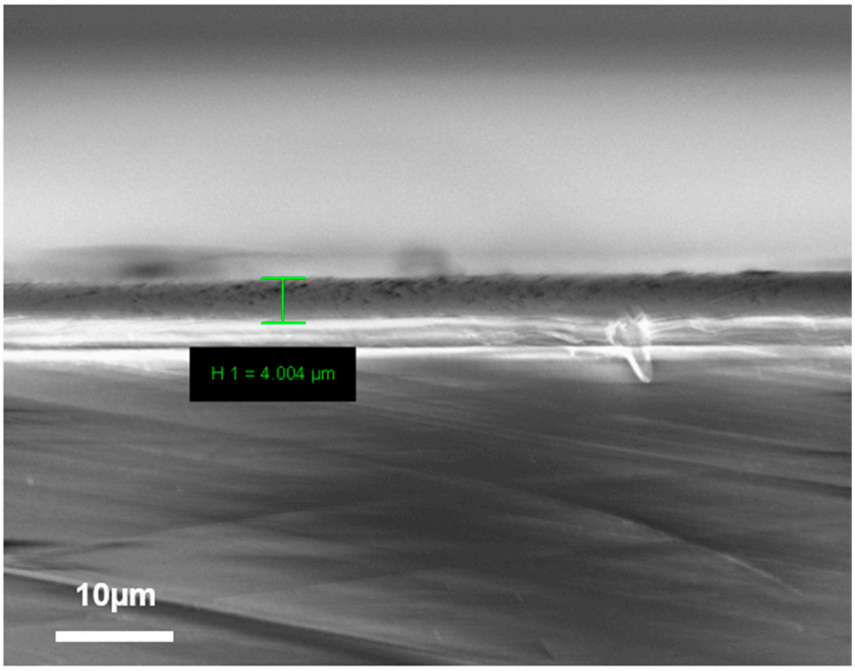

Figure S5. The cross-sectional SEM image of the photochromic film (Thickness is about 4 ~ 5 μm).

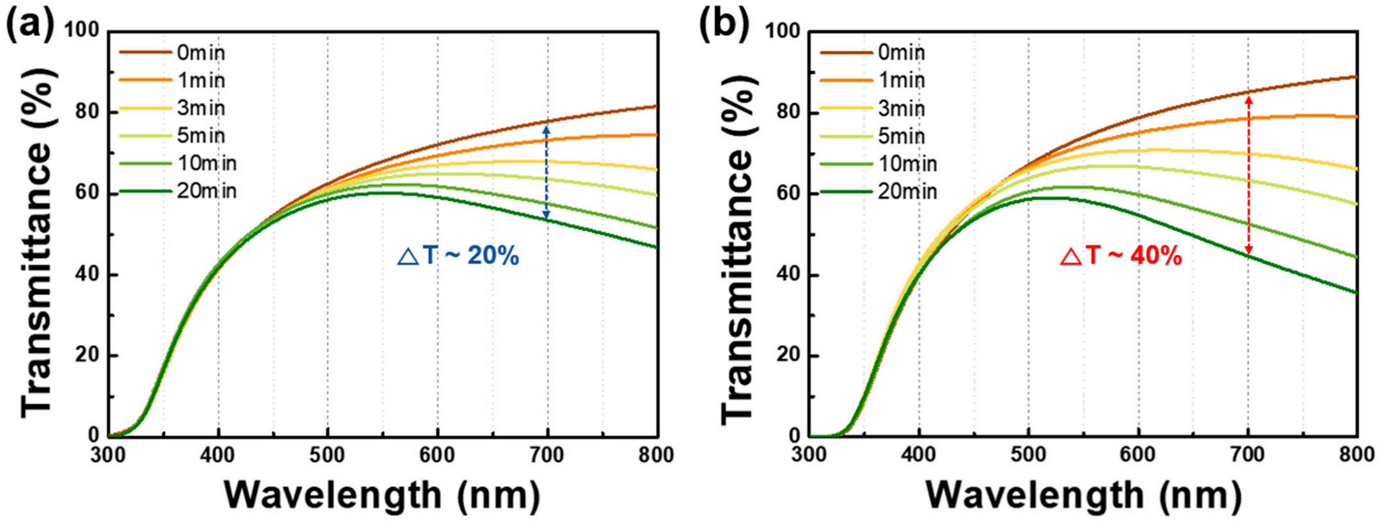

Figure S6. Transmittance of photochromic films. (a) WO_3@PVP composite, (b) WO_3/TiO_2@PVP composite. The introduction of titanium oxide resulted in an improvement of photochromic properties by more than twofold at the 700 nm wavelength.

Reference

1. Khan, H.; Rigamonti, M.G.; Patience, G.S.; Boffito, D.C. Spray dried TiO2/WO3 heterostructure for photocatalytic applications with residual activity in the dark. *Applied Catalysis B: Environmental* **2018**, *226*, 311-323.
2. Evdokimova, O.; Kusova, T.; Ivanova, O.; Shcherbakov, A.; Yorov, K.E.; Baranchikov, A.; Agafonov, A.; Ivanov, V. Highly reversible photochromism in composite WO 3/nanocellulose films. *Cellulose* **2019**, *26*, 9095-9105.
3. Krasnikov, I.; Popov, A.; Seteikin, A.; Myllylä, R. Influence of titanium dioxide nanoparticles on skin surface temperature at sunlight irradiation. *Biomedical Optics Express* **2011**, *2*, 3278-3283.
4. Liao, Y.; Lin, J.; Cui, B.; Xie, G.; Hu, S. Well-dispersed ultrasmall ruthenium on TiO2 (P25) for effective photocatalytic N2 fixation in ambient condition. *Journal of Photochemistry and Photobiology A: Chemistry* **2020**, *387*, 112100.
5. Liao, Y.; Qian, J.; Xie, G.; Han, Q.; Dang, W.; Wang, Y.; Lv, L.; Zhao, S.; Luo, L.; Zhang, W. 2D-layered Ti3C2 MXenes for promoted synthesis of NH3 on P25 photocatalysts. *Applied Catalysis B: Environmental* **2020**, *273*, 119054.
6. Wang, Y.; Wang, X.; Xu, Y.; Chen, T.; Liu, M.; Niu, F.; Wei, S.; Liu, J. Simultaneous Synthesis of WO(3-)(x) Quantum Dots and Bundle-Like Nanowires Using a One-Pot Template-Free Solvothermal Strategy and Their Versatile Applications. *Small* **2017**, *13*, doi:10.1002/smll.201603689.
